# Extracting biologically significant patterns from short time series gene expression data

**DOI:** 10.1186/1471-2105-10-255

**Published:** 2009-08-20

**Authors:** Alain B Tchagang, Kevin V Bui, Thomas McGinnis, Panayiotis V Benos

**Affiliations:** 1 Department of Computational Biology, University of Pittsburgh, Pittsburgh, PA 15260, USA; 2 Department of Computer Science, University of Pittsburgh, Pittsburgh, PA 15260, USA

## Abstract

**Background:**

Time series gene expression data analysis is used widely to study the dynamics of various cell processes. Most of the time series data available today consist of few time points only, thus making the application of standard clustering techniques difficult.

**Results:**

We developed two new algorithms that are capable of extracting biological patterns from short time point series gene expression data. The two algorithms, *ASTRO *and *MiMeSR*, are inspired by the *rank order preserving *framework and the *minimum mean squared residue *approach, respectively. However, *ASTRO *and *MiMeSR *differ from previous approaches in that they take advantage of the relatively few number of time points in order to reduce the problem from NP-hard to linear. Tested on well-defined short time expression data, we found that our approaches are robust to noise, as well as to random patterns, and that they can correctly detect the temporal expression profile of relevant functional categories. Evaluation of our methods was performed using Gene Ontology (GO) annotations and chromatin immunoprecipitation (ChIP-chip) data.

**Conclusion:**

Our approaches generally outperform both standard clustering algorithms and algorithms designed specifically for clustering of short time series gene expression data. Both algorithms are available at .

## Background

Time series experiments have been widely used to study the dynamic behavior of the cells in a variety of biological processes, including cell proliferation [1], development [2], and response to extracellular *stimuli *[3,4]. Time series data can be broadly divided into two classes: the *short-time series *with few sampled time points (typically 3–8) and *long-time series *with more than 10 time points sampled. Most algorithms used to analyze time series datasets initially were based on general clustering methods like hierarchical clustering [5], *k*-means [6], Bayesian networks [7], and self-organizing maps [8]. Although these methods are capable of revealing some biological features, they are not taking into consideration the sequential nature of the time series data. More recently, some groups suggested methodologies specifically designed for clustering time series expression data, including the use of continuous representation of expression profiles [9], hidden Markov models [10], and others [11-14]. However, algorithms such as those developed by Bar-Joseph *et al*. [9], De Hoon *et al*. [12] and Peddada *et al*. [13] perform better on long time series datasets where the statistical power is higher. For short time series data, which represent about 80% of the time series gene expression datasets [15], they are expected to perform less optimal due to data overfitting caused by the small number of sampled time points.

In order to avoid that, some researchers have suggested the use of predefined patterns of expression profiles (either taken directly from the data or from prior biological observations) and matching the observed data to these profiles using some cost function [15-18]. Such approaches usually identify a large number of patterns, but many of them may arise randomly from noise due to the small number of sampled time points. The algorithm proposed by Ernst *et al*. [15] is capable of partially correcting for this problem with the implementation of heuristics: the user is required to select a set of potential profiles that are expected to represent better the real biological nature of such data. Last but not least, almost all of the approaches mentioned above use a cost function followed by a greedy algorithm to find clusters. As we will show later, such approaches may miss some biologically significant characteristics of the data.

In this paper, we present two new algorithms, *ASTRO *and *MiMeSR*, respectively, which are specifically designed to identify biologically relevant clusters of genes from short time series data. *ASTRO *and *MiMeSR *are inspired by the *order preserving *framework and the *minimum mean squared residue *approach, respectively. Other algorithms have used the same principles in the past, but in the biclustering context [19-21], which makes such algorithms *NP *hard [21]. We demonstrate the utility of *ASTRO *and *MiMeSR *using several well-defined short time datasets. We show that our approaches are robust to noise and random patterns and they can correctly detect the temporal expression profile of relevant functional categories in linear time. Comparative analysis also showed that our approaches outperform both general clustering algorithms and algorithms designed specifically for short time series gene expression data.

## Results and Discussion

### Robustness to noise

**Figure 1:**
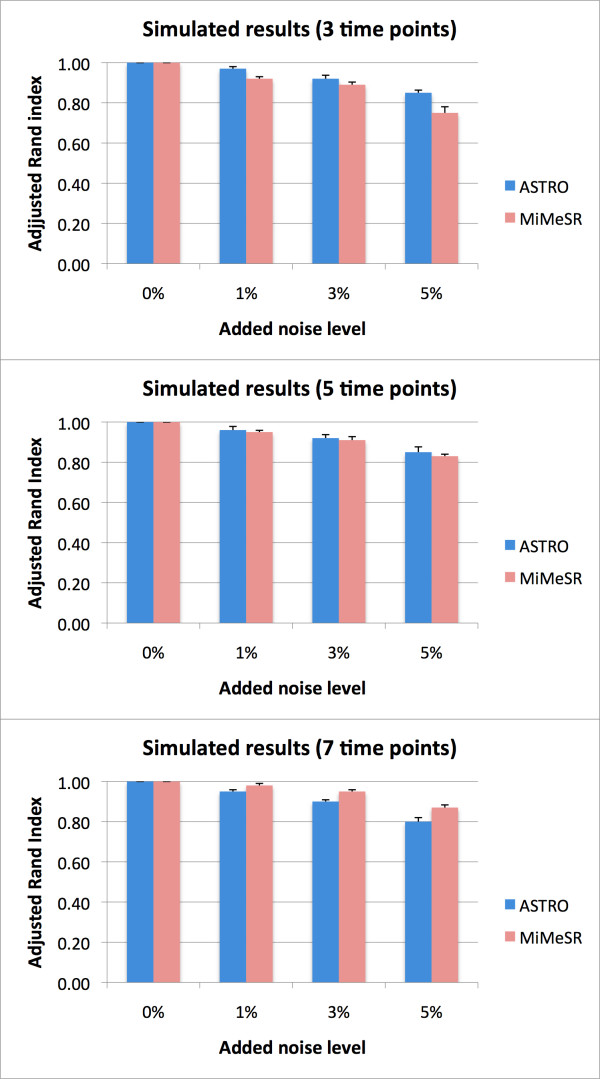
***ASTRO *and *MiMeSR *performance on simulated data**. Comparison of the two algorithms on simulated data with different noise levels.

To test the robustness of *ASTRO *and *MiMeSR *to noise, we generated three sets of data, 1000 rows and 3, 5, and 7 time points respectively, with five order preserving submatrix which at the same time verify the minimum mean squared residue property embedded in it (domain knowledge). Then, we added 0%, 1%, 3%, and 5% level of noise into the simulated data. We ran each algorithm several times on each set of data and plot the average of the Adjusted Rand index (Figure 1). The Adjusted Rand index values lies between 0 and 1. Larger value means higher similarity between the clustering results. If the simulated result is perfectly consistent to the domain knowledge, the index value will be 1. If a clustering is no more than a random choice, the index will be zero [22]. The results in Figure 1 show that both algorithms perform equally well on the 5 time points dataset, while ASTRO is more robust on the 3 time points datasets and *MiMeSR *on the 7 time points dataset.

### Application on Saccharomyces cerevisiae amino acid starvation dataset

We tested the ability of *ASTRO *and *MiMeSR *to identify biologically relevant clusters from short time series data using the yeast amino acid (AA) starvation dataset [3]. *Saccharomyces cerevisiae *response to stress by AA starvation is measured at time points 0.5 h, 1 h, 2 h, 4 h, and 6 h and at the control (unstimulated) cells (time point 0 h). The data was filtered to remove genes with missing values and genes whose expression level did not change substantially between time points, filtering threshold *ε < 2.0 *for *ASTRO *and *MiMeSR*. The results show that both our approaches can correctly identify the temporal profiles of relevant functional groups. Statistical evaluation of our clusters was performed using external datasets, like the GO categories [23] and AA starvation ChIP-chip data [3,24]. Compared to general clustering algorithms (e.g., *k*-means) and algorithms designed specifically for clustering short time series gene expression data [17,25], our techniques were able to detect more significant patterns.

#### Evaluation using GO annotations

**Figure 2:**
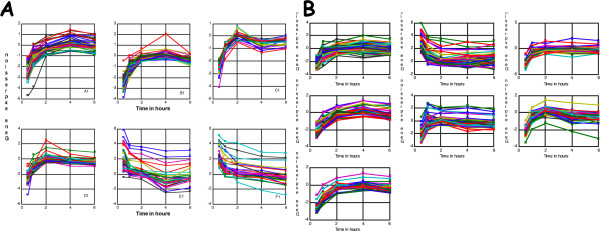
**Clustering yeast time series data**. The most statistically significant clusters as they were identified by **(A) ***ASTRO *and **(B) ***MiMeSR*.

Figure 2A and 2B present the plot of the most significant clusters identified in this dataset by *ASTRO *(*Z = 10^-7 ^to 10^-68^*) and *MiMeSR (H < 2)*, respectively. The minimum number of genes per cluster in both cases was set to K_min _= 25. In principle, one might expect that the genes in biologically relevant clusters will also participate in the same biological processes. We used the on-line yeast GO Term Finder tool  to assess GO membership of the genes in the identified clusters. We found that with the exception of the clusters with many genes of unknown function, the majority of the genes in the identified clusters belong to the same GO categories. The *p*-values for these clusters were ranging from 10^-10 ^to 10^-34 ^for *ASTRO *(Table 1) and from 10^-34 ^to 10^-68 ^for *MiMeSR *(Table 2.) The results also show that in general *MiMeSR *clusters are more homogenous than the *ASTRO *clusters regarding the GO pathways. For example, the percentage of the genes in the *ASTRO *clusters C1 and D1 that belong to the "*ribosome biogenesis*" category (Table 1) is smaller than *MiMeSR *clusters E2 and F2 (Table 2); consequently, their *p*-values are higher. The same is true for *ASTRO *cluster B1 and *MiMeSR *clusters D2 and G2.

**Table 1:** Evaluation of the clusters identified by *ASTRO *using Gene Ontology data

***ASTRO (OPSM)***				
**Clusters**	**No. of genes**	**Top GO term**	**# of genes in category**	***p*-value**

A1	70	Cellular biosynthetic process	41 (58.6%)	7.0e-16
		Biosynthetic process	42 (60.0%)	2.5e-13

B1	86	Translation	55 (64.0%)	1.5e-33
		Macromolecule biosynthetic process	55 (64.0%)	1.5e-27

C1	25	Ribosome biogenesis and assembly	14 (56.0%)	9.8e-15
		Ribonucleoprotein complex biogenesis and assembly	14 (56.0%)	9.6e-10

D1	74	Ribosome biogenesis and assembly	44 (59.5%)	2.6e-34
		Ribonucleoprotein complex biogenesis and assembly	44 (59.9%)	4.6e-31

E1*	33	Sulfur metabolic process	06 (18.2%)	9.0e-05
		Sulfur amino acid metabolic process	04 (12.1%)	2.4e-03

F1*	26	Amino acid transport	04 (15.4%)	1.4e-03
		Amine transport	04 (15.4%)	3.6e-03

* More than 20% genes of unknown function

**Table 2:** Evaluation of the clusters identified by *MiMeSR *using Gene Ontology data

***MiMeSR (MMSR)***				
**Clusters**	**No. of genes**	**Top GO term**	**# of genes in category**	***p*-value**

A2	246	Ribosome biogenesis and assembly	103 (42.0%)	2.0e-64
		Gene expression	167 (68.0%)	1.0e-49

B2*	66	Sulfur metabolic process	13 (20.0%)	1.2e-12
		Sulfur amino acid metabolic process	8 (12.1%)	4.4e-08

C2	133	Ribosome biogenesis and assembly	82 (62.0%)	1.7e-68
		Ribonucleoprotein complex biogenesis and assembly	84 (63.2%)	5.6e-65

D2	80	Translation	62 (77.5%)	1.5e-46
		Macromolecule biosynthetic process	66 (82.5%)	1.9e-45

E2	109	Ribosome biogenesis and assembly	73 (67.0%)	1.7e-64
		Ribonucleoprotein complex biogenesis and assembly	73 (67.0%)	8.1e-59

F2	60	Ribosome biogenesis and assembly	42 (70.0%)	1.6e-37
		Ribonucleoprotein complex biogenesis and assembly	42 (70.0%)	2.2e-34

G2	94	Translation	76 (81.0%)	3.1e-60
		Macromolecule biosynthetic process	77 (82.0%)	5.2e-53

* More than 20% genes of unknown function

#### Evaluation using ChIP-chip data

Another reasonable assumption is that genes that belong to the same cluster (co-expressed genes) are more likely to be regulated by the same transcription factors (co-regulated genes). We evaluated the *ASTRO *and *MiMeSR *clusters using the published AA starvation ChIP-chip dataset on 34 transcription factors [24]. Each of the 34 transcription factors in the ChIP-chip dataset was tested for target over-representation in each of the clusters using the *Fisher's exact test*. The results are presented on Tables 3 (*ASTRO*) and 4 (*MiMeSR*). FHL1, and SFP1 appear to have overrepresented number of target genes in clusters *A1*, *B1*, *D1 *(Table 3), *A2*, *C2*, *D2*, and *G2 *(Table 4), which are also the most similar in their overall expression pattern (Figure 2A, 2B), especially in 0.5 hr and 1 hr time points. It is possible that these transcription factors act early on in the AA starvation response as it was previously suggested [26]. Consistent with our results, Jorgensen *et al*. [27] have found that FHL1 and SFP1 regulate many ribosome biosynthesis genes, which is the most significant GO process in cluster *C1*, *D1*, *A2*, *C2*, *D2*, *E2*, and *F2 *genes. Furthermore, MET31, MET32 and MET4 have been associated with regulation of sulfur metabolism genes [28], which is the most significant category for our cluster *E1 *genes.

**Table 3:** Evaluation of the clusters identified by *ASTRO *using ChIP-chip data.

	**ASTRO (OPSM)**					
**TFs**	**A1**	**B1**	**C1**	**D1**	**E1**	**F1**

ARO80	2/3% (5e-02)	3/3% (7e-02)				1/4% (4e-02)

BAS1					1/3% (8e-02)	

CBF1					5/15% (1e-02)	

CHA4				**4/4%** **(9e-03)**		

DAL81						**1/4%** **(4e-03)**

FHL1	**25/36%** **(3e-20)**	**43/50%** **(3e-42)**	3/10% (1e-02)	**19/25%** **(1e-11)**		

GCR2	3/4% (2e-02)					

GCN4					3/9% (7e-02)	

MET31					1/3% (7e-02)	

MET32					**4/12%** **(6e-12)**	

MET4					1/3% (7e-02)	

SFP1	**6/9%** **(1e-05)**	**13/15%** **(2e-14)**	3/10% (1e-02)	**8/11%** **(5e-08)**		

**Bold letter **boxes correspond to statistically overrepresented transcription factors in that cluster. Each box contains: (a) the number of genes, (b) the percent of genes in the cluster associated with this transcription factor, and (c) the *p*-value (*Fisher's exact test*).

**Table 4:** Evaluation of the clusters identified by *MiMeSR *using ChIP-chip data.

	**MiMeSR (MMSR)**						
**TFs**	**A2**	**B2**	**C2**	**D2**	**E2**	**F2**	**G2**

ARO80			4/3% (3e-02)				

BAS1							

CBF1	10/4% (1e-02)	**9/14%** **(3e-03)**			3/3% (1e-02)		

CHA4							

DAL81							

FHL1	**86/35%** **(3e-20)**		**27/20%** **(7e-14)**	**48/60%** **(1e-52)**	5/5% (1e-02)	3/5% (7e-02)	**62/65%** **(2e-70)**

GCR2				**4/5%** **(5e-03)**			

GCN4							

MET31							

MET32		**4/5%** **(9e-03)**		3/3% (9e-02)			

MET4							

SFP1	**30/12%** **(1e-08)**		4/3% (1e-02)	**17/21%** **(1e-21)**			**19/20%** **(1e-20)**

**Bold letter **boxes correspond to statistically overrepresented transcription factors in that cluster. Each box contains: (a) the number of genes, (b) the percent of genes in the cluster associated with this transcription factor, and (c) the *p*-value (*Fisher's exact test*).

Comparison of the results in Tables 3 and 4 also shows that in general *ASTRO *finds more clusters that are statistically significantly enriched in genes bound by transcription factors. In other words, *ASTRO *performs better than *MiMeSR *in identifying *co-regulated *genes.

#### Comparison with other methods

**Figure 3:**
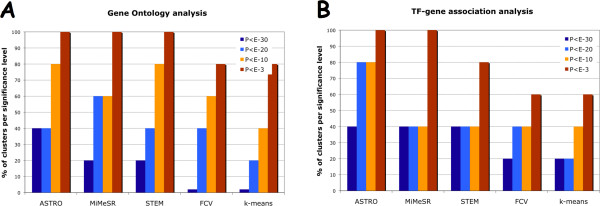
**Comparison of clustering approaches**. Comparative analysis of clustering approaches using **(A) **GO data and **(B) **amino acid starvation ChIP-chip data. The y-axis represents the percent of clusters for which the *p*-value of their most significant category (GO or ChIP-chip) was lower than the given threshold.

We compared *ASTRO *and *MiMeSR *with the popular *k*-means general clustering algorithm and the recently published STEM [25] and FCV [17], both of which are designed specifically for short time series gene expression data. We used the Matlab 7.0.0 implementation of ***k*-means **with correlation distance. We ran the *k*-means algorithm for 10 clusters because most of our algorithm picked the same number. **FCV **was implemented according to the description provided in [17]. **STEM **ran over the web  with the following parameters: maximum unit of change in model profiles between time points = 4; number of model profiles = 50. For unbiased comparison, we ran these algorithms on the same dataset of 698 genes (filtering threshold *ε < 2*). We selected clusters with less than 50% genes of unknown function as depicted by the GO database. Figure 3A and 3B presents the comparative evaluation of these approaches using GO and ChIP data, respectively. A particular cluster was considered to be "*significant*" if the *p*-value of the top GO category or transcription factor-gene association was smaller than the threshold. A good clustering algorithm is expected to identify sets of genes that will participate in the same biological processes (GO annotation) and/or regulated by the same transcription factors. The more homogeneous these clusters are the more significant the annotation categories will become. *ASTRO *identified a higher number of significant clusters than the *k*-means and the FCV algorithms in *all p*-value thresholds (Figure 3A and 3B). Also, it performed equally well or better when compared to STEM. *MiMeSR *also performed equally well or better than all other algorithms with respect to the TF-gene association data; and it gave comparable results to the other algorithms in the GO category analysis. In particular, we found it to be more accurate than the other algorithms in predicting the more tight clusters (low *p*-values), whereas the other algorithms performed better in higher *p*-values. This shows the potential of our algorithms in identifying more tight, biologically relevant clusters. We note, however, that a thorough comparison of different methods is impossible when dealing with noisy datasets and algorithms with different reporting thresholds.

## Conclusion

In this study, we presented two new algorithms for analyzing short time series gene expression data: *ASTRO *that uses the *order preserving matrix *concept and *MiMeSR *that uses the *minimum mean squared residue *concept. Both these algorithms use linear algebra techniques to identify coherent gene clusters over all time points in linear time. This offers a significant advantage over existing methods that employ greedy approaches or heuristics, since *ASTRO *and *MiMeSR *avoid problems related to cost functions or the choice of predefined sets of expression profiles [19,21,25]. Also, the complexity of our methods is smaller than that of existing algorithms, even when it is compared to those that use a greedy approach to speed up their running time [9-15]. *ASTRO *identifies all gene clusters with coherent expression patterns, irrespectively of the magnitude of expression change. Genes belonging in such clusters are generally expected to participate in the same biological processes (see, also, Table 1). *MiMeSR *identifies all gene clusters with coherent expression change both in direction as well as in magnitude. Genes belonging in such clusters are expected to be regulated by the same set of transcription factors (although different transcription factors may act at different time points). *In general, MiMeSR identifies clusters that are enriched for genes belonging to the same pathways, while ASTRO identifies clusters with genes that are regulated by the same transcription factors *(Tables 1, 2, 3 and Figure 3). In terms of overall statistical significance of the identified clusters, *MiMeSR *outperforms *ASTRO *in most cases (Figure 3). In a direct comparison of the two algorithms in simulated data (Figure 1) we found that *ASTRO *outperforms *MiMeSR *when fewer data points are available, whereas *MiMeSR *performs better when more data points are available.

Testing *ASTRO *&*MiMeSR *in well-characterized short time series gene expression datasets showed that it is robust to noise and to random patterns, and that it can correctly predict the temporal expression profile of relevant functional categories as confirmed by statistical analysis of GO category membership over-representation and analysis of transcription factor occupancy in the promoters of the gene members of the various clusters. Our approaches are shown to outperform existing clustering algorithms, including the popular *k*-means, as well as FCV and STEM and they were able to distinguish between closely related but biologically distinct patterns. As expected, *ASTRO *finds more homogeneous clusters than *MiMeSR*, with respect to the percentage of genes associated with a given transcription factor. This is because it takes into consideration the magnitude of the change in gene expression, which is more closely related to the transcription factors involved.

In principle, *ASTRO *and *MiMeSR *can also be applied to long time series gene expression data (more than 10 time points) or gene expression data sampled over different conditions, but in this case the number of genes in each cluster is expected to be low. However, they can be adapted to identify local patterns, thus overcoming this problem.

## Methods

### General description of the algorithms

A time series gene expression dataset can be represented by an *N × M *matrix, *A *= *[a_nm_]*, with rows corresponding to the genes from *G *= *{g_1_,..., g_n_,..., g_N_}*, and columns corresponding to the time point measurements from *T *= *{t_1_,..., t_m_,..., t_M_}*. The entry *a*_*nm *_is the expression level of gene *n *at time point *t*_*m *_or -simply- *m*. Given a short time-series gene expression dataset, our goal is to identify the sets of genes with coherent behavior, *i.e*., genes whose expression levels increase and/or decrease coherently across the time point experiments, by minimizing the effect of noise and random patterns. The input data are pre-processed to identify and remove from the matrix all genes whose expression level remains constant across time points. We consider the expression of a gene to be constant when the difference between the minimum and the maximum value (in log-scale) is less than a positive real number, *ε*. *ε *is a user-defined parameter and it can be based on prior knowledge on the expected level of noise on a given experiment.

### ASTRO (Rank Order Preserving Matrix framework)

*ASTRO *seeks to identify sets of genes with similar expression profiles irrespectively of their expression fold-change. Therefore, only the direction of expression change is considered and not its magnitude. This reflects the biological fact that different gene products may be required at different quantities for a given cellular response or function. Under this assumption, *the problem of finding a cluster of similarly expressed genes can be caste into a problem of finding an order preserving submatrix (**OPSM**) of the gene expression matrix A*. A submatrix *C *of *A *is *order preserving (OP) *if there is a permutation of its columns under which the sequence of values in every row is strictly increasing or decreasing. In other words an OPSM is a set: *C *= *{2-tuples (I,J), I ∈ G and J ∈ T}*, such that each row induces the same rank order permutation on the columns. The problem of searching over all possible subsets of columns for identifying the most significant OPSM is *NP *hard [19]. However, taking advantage of the small number of sampled points in a short time series dataset, one may seek patterns of consistent gene expression over *all *time points. In such case the order will be required to be preserved in *all *columns (time-points) for the genes in a cluster (*i.e. J = T*). In fact, this assumption is now commonly used in analyzing short-time series experiments [9-15]. As we show below, this reduces the complexity of finding OPSMs, which offers an advantage over methods that use probabilistic models and greedy algorithms. *ASTRO *is guaranteed to find all OPSMs across all time points in O(NM) using linear algebra techniques.

#### Overview

In this part, we focus on genes with coherent behavior, *i.e*., genes whose expression levels increase and/or decrease coherently across all time points. *ASTRO *starts by filtering those genes with constant gene expression across all time points (using a threshold *ε*). It then proceeds by constructing the rank matrix of the time series gene expression data. Next, it identifies all distinct coherent patterns in the ranked matrix. Finally, it assigns each gene to its corresponding cluster by performing a row comparison between the set of distinct rows of the ranked matrix and the ranked matrix itself. See Additional file 1 for *ASTRO *pseudo codes.

#### Rank Matrix Construction

A *rank matrix *is an *N × M *matrix, *R *= *[r_nm_]*, in which every row (gene) is a vector of the *ranks *of the corresponding expression values in *A *in increasing order. For example, if the expression levels of gene *g*_*i *_are *A*_i* _= (5, 10, 15, 8) then the corresponding row in the rank matrix would be *R*_i* _= (1, 3, 4, 2). The ranking is performed in increasing order. If more than two entries have the same value, the user can decide to give them the same ranking or rank them in the order they appear. In this study, we choose the former. By replacing each entry of the gene expression matrix with their rank along the rows, we are no longer considering the expression level of a given gene *per se*, but its dynamics over all time points. The advantage of this method is its speed and that it avoids the use of probabilistic models, greedy algorithms, or costly column permutations. Also, one will notice that for any *k > 1 *rows of the ranked matrix that are similar under any permutation of the columns of the gene expression matrix; they will always belong to the same cluster. Finally, the fact that the rank is conserved under any permutation of the columns of the gene expression matrix further reduces the chance that a random pattern might be picked up in a cluster (see, also, the *Statistical Significance and Complexity Analysis *section).

#### Pattern Identification

Given a gene expression data matrix, *A*, the exact number of distinct OP expression profiles that can be found in the dataset (time points *t *= *1,..., M*) is the number of distinct rows, *N*_*U*_, of its corresponding ranked matrix *R*. The set of distinct OP patterns, *U*, can thus be identified by considering the rank matrix *R *as a set of rows and identify all subsets of identical rows in it. *ASTRO *is guaranteed to identify the exact number of distinct OP patterns in a given matrix in O(NM).

#### Identification of Order Preserving Clusters

Once the exact number of distinct OP patterns has been identified, *ASTRO *assigns each gene to one of the *N*_*U *_groups by comparing each distinct row *U*_k* _of the ranked matrix to the rows *R*_n* _of the ranked matrix itself, and assign gene *n *to cluster *G{k} *each time *U*_k* _= *R*_n*_. This approach is guaranteed to identify all OP clusters of size *K × M*, with *K*_*min *_≤ *K *≤ *N*, and *K*_*min *_is the minimum number of genes in a cluster.

#### Statistical Significance and Complexity Analysis of ASTRO

The statistical significance of each identified cluster with *K *genes is assessed by computing the tail probability that a random dataset of size *N × M *will contain a cluster with *K *or more genes in it [19]. In principle, the probabilistic description of the reference random matrix would be that of the observed noise in the microarray experiment [29,30]. Since this distribution is difficult to calculate in closed form, we calculate the upper bound of this tail probability following the approach described below. Let's assume we have a dataset with *M *time points that are independent, identically distributed according to the uniform distribution. Then the probability that a random row (gene) supports a given cluster is equal to the number of possible column permutations or *1/M!*. Since the rows are assumed to be independent, the probability of having at least *K *rows in the cluster is the *k*-tail of the (N,(1/M!)) binomial distribution, *i.e*.:



As there are *M*_*s *_= *M! *ways to choose an OP cluster of size *M*, the following expression *Z(M,K) *is an upper bound on the probability of having a cluster of size *M *with *K *or more genes:



We use this bound to estimate the significance of any given cluster of size *M *with *K *members. The best cluster is the one with the largest statistical significance, *i.e*., the one with the *smallest Z(M,K)*. Therefore, as long as that upper bound probability is smaller than any desired significance level, the identified cluster in the real gene expression matrix will be statistically significant.

The overall complexity of *ASTRO *is ~*O(NM)*. Recall that the time series gene expression *A *is an *N × M *matrix. The rank matrix can be identified with an *O(NM) complexity*. The number of distinct OP patterns and the set of distinct OP patterns can be identified with a complexity less than *O(N)*. Finally, clusters can be identified with a complexity less than *O(N)*. In all, the complexity of *ASTRO *is *O(NM) + O(N) + O(N)*, which is ~*O(NM)*, less that the complexity of existing approaches.

### MiMeSR (Minimum Mean Squared Residue)

*MiMeSR *seeks to solve the same problem, but unlike *ASTRO*, it takes into consideration the *magnitude *of the expression change in the analysis. This is based on the (biological) assumption that if a set of genes is regulated by the same transcription factors across all time points (even if different transcription factors are active at different time points,) then the expression pattern of these genes will not only be the same in terms of direction, but also in magnitude. In other words, *MiMeSR *aims to identify more coherent clusters of *co-regulated *genes rather than simply genes with similar expression patterns under a given set of conditions. Under this hypothesis, *the problem of finding a cluster of similarly expressed genes is a problem of finding submatrices of the gene expression matrix, A*, with *minimum mean squared residue *or coherent values [20,21]. A cluster here is defined as a submatrix *C *= *[c_ij_] *of *A *(with *i *and *j *correspond to the gene and time point, respectively), such that its mean squared residue *H(C) < δ*. The mean squared residue *H(C) *of *C *is computed using the following formula:



where *c*_*iT *_is the mean of the *i*^*th *^row (expression of gene *i *over all time points), *c*_*Gj *_is the mean of the *j*^*th *^column (expression of all genes at the *j *time point) and *c*_*GT *_is the mean of all the elements of *C*. When *C *= *[c(i,j)] *= *[a_i _+ b_j_] *= *[a_i_] + [b_j_]*, where *[a_i_] *a matrix with constant values on rows, and *[b_j_] *a matrix with constant values on columns, then it can be shown that the mean squared residue, *H(C)*, of *C *is zero.

*Proof*.



The *MiMeSR *algorithm that we develop in this study uses this concept to search for submatrices with mean squared residue smaller that a given threshold, ***δ → 0***.

Cheng and Church have shown that when the search extends over all possible subsets of columns, then the solution is *NP *hard [20]. As we will show below, looking for patterns consistent over *all *time points (*i.e*. J = T) reduces the algorithmic complexity to O(NMK), and produces biologically relevant results. *MiMeSR *uses linear algebra and arithmetic tools to solve the problem, which is advantageous over greedy algorithms or the use of heuristics that were used in the past.

#### Overview

*MiMeSR *starts by filtering those genes whose expression levels do not change significantly during the time course (threshold ***ε***). Then, it writes the gene expression matrix *A *as the sum of matrix *Z*_1_, with constant values on columns, and *Z*_2 _= *A *- *Z*_1_. Finally, it identifies submatrices with constant values on rows in *Z*_2_, which correspond to the *minimum mean squared residue clusters *in the gene expression matrix *A*. See Additional file 1 for *MiMeSR *pseudo codes.

#### Identification of minimum mean squared residue clusters

**Figure 4:**
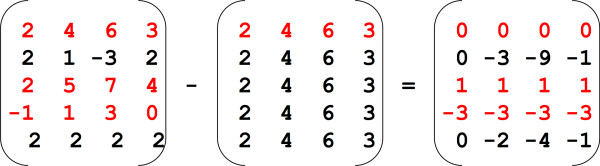
**Example of the *minimum mean squared residue *method**.

*MiMeSR *extracts *minimum mean squared residue submatrices *from the gene expression matrix using the following approach. For a given row *i *of matrix *A*, a new matrix *Z*_1 _is constructed with constant values in the columns. All rows in *Z*_1 _are identical to row *A[i]*. Then *Z*_2 _is calculated as *Z*_2 _= *A *- *Z*_1_. Then, *MiMeSR *identifies the submatrix with constant values on rows across the whole time points in *Z*_2_. This step is easily performed by identifying the set of rows of *Z*_2 _such that *max(Z_2_(n,:) - min(Z_2_(n,:)) <**ε***, with *ε → 0*. The submatrices with constant values on rows in *Z*_2 _correspond to submatrices with *minimum mean squared residue *(coherent values) in *A*. For simplicity and without loss of generality, let us consider an example of the synthetic gene expression matrix *A*, with coherent values cluster in it, corresponding to rows *r*_1_, *r*_3 _and *r*_4 _(Figure 4.) By subtracting from *A *matrix *Z*_1 _(constructed using the first row of *A, (2 4 6 3)*), a new matrix *Z*_2 _is generated whose rows *r*_1_, *r*_3_, and *r*_4 _correspond to the submatrix with constant values on rows. Note that, the same cluster will be constructed by using any of the rows *r*_1_, *r*_3_, or *r*_4_. Therefore, after a cluster has been identified, its rows are not further considered in the construction of new *Z*_1 _matrices. This approach is guaranteed to identify all submatrices with *minimum mean squared residue *across all time point experiments. Note that, since the operation *Z*_2 _= *A *- *Z*_1 _is performed using all the rows of *A *during each iteration, and since we are seeking for the set of rows of *Z*_2 _such that *max(Z_2_(n,:) - min(Z_2_(n,:)) < ε*, *MiMeSR *can allow rows (genes) to belong to more than one cluster. The biological equivalent of this notion is that genes may be involved in more than one genetic pathway or to be regulated by more than one transcription factors.

#### Statistical Significance and Complexity Analysis of MiMeSR

For practical reasons, it is important to assess the effects of the *ε *parameter on the clusters that are identified by *MiMeSR*. This can be done by *sensitivity analysis *in which the parameter *ε *is perturbed and the results are compared. For this analysis, it is usually sufficient to consider one or two values above and below the originally selected value of *ε*. Only clusters that are consistently identified by *MiMeSR *as *ε *varies should be retained for further examination. Note that the number of genes in these clusters may also change. The user therefore needs to determine a rule for dealing with genes that may be dropped from the clusters as *ε *changes. The most conservative approach would be to retain only the genes that remain in the clusters for all values of *ε *around its selected value. It can be easily shown that the overall complexity of *MiMeSR *is ~*O(NMK)*, where *K *is the number of minimum mean squared residue clusters in *A*. Note that *K *corresponds to the maximum number of constant columns matrices that can be constructed using the rows of *A *without identifying redundant clusters.

## Authors' contributions

PVB and ABT designed the study, analyzed the results and wrote the paper. KVB and TM designed the web server and contributed to the writing of the paper.

## Supplementary Material

Additional file 1 **Supplementary materials**. The following additional data are available with the online version of this paper. Additional file 1 contains the pseudo codes for *ASTRO *and *MiMeSR*. Click here for file
