# Supplementary material for: Extracting biologically significant patterns from short time series gene expression data
Source: BMC Bioinformatics. 2009 Aug 20;10:255. doi: 10.1186/1471-2105-10-255 (PMC2743670; doi:10.1186/1471-2105-10-255)
Supplement: Additional file 1 — Supplementary materials. The following additional data are available with the online version of this paper. Additional file 1 contains the pseudo codes for ASTRO and MiMeSR. [file 1471-2105-10-255-S1.pdf]

SUPPLEMENTARY MATERIALS for the paper

Alain B. Tchagang, Kevin V. Bui, Thomas McGinnis, Panayiotis V. Benos,
"***Extracting biologically significant patterns from short time series gene expression data***"

ASTRO pseudo code

Input

- A_0 = raw gene expression data,
- G = set of genes
- T = set of sample time points
- ε = filtering threshold

Output

- *C{k}* = coherent clusters
- *I{k}* = set of genes in clusters
- *J{k}* = set of time points in clusters

Begin

```
A = filter(A0, ε); [N,M] = size(A); % remove constant genes.
R = rank(A); [NR,MR] = size(R); % rank matrix construction.
U = union(R,R,'rows'); [NU,MU] = size(U); % identify sets of distinct patterns.
    For k = 1:NU
        For n = 1:NR
            If R(n,:) == U(k,:)
                C{k} = [C{k};A(n,:)]
                I{k} = [I{k};G(n)];
            End If
        End For; J{k} = T;
    End For
End begin
```

MiMeSR pseudo code

Input

- A_0 = raw gene expression data,
- G = set of genes
- T = set of sample time points
- ε = filtering threshold
- I_0 = minimum number of genes in a cluster

Output

- $C\{k\}$ = minimum mean squared residue clusters
- $I\{k\}$ = set of genes in clusters
- $J\{k\}$ = set of time points in clusters

Begin

A = ***filter****(A*$_0$*, ε); [N,M] =* ***size****(A); k = 0; X = [0];*

For *n = 1:N*

If $n \notin X$

- Construct Z_1, using row *n of A*.
- $Z_2 = A - Z_1$
- *Index =* ***find(max****(Z*$_2$*')-****min****(Z*$_2$*') < ε)*
- ***If*** $Index \geq I_0$
  - *k = k+1;*
  - *C{k} = A(Index,:);*
  - *I{k} = G(Index);*
  - *J[k} = T;*
- ***End If***
- *X =* ***union****(X,Index)*

End If

End For

End begin
